# Integrating stay-green and PIN-FORMED genes: PIN-FORMED genes as potential targets for designing climate-resilient cereal ideotypes

**DOI:** 10.1093/aobpla/plad040

**Published:** 2023-07-06

**Authors:** Albert Chern Sun Wong, Erik J van Oosterom, Ian D Godwin, Andrew K Borrell

**Affiliations:** Queensland Alliance for Agriculture and Food Innovation (QAAFI), The University of Queensland, 306 Carmody Road, Brisbane, Queensland 4072, Australia; Queensland Alliance for Agriculture and Food Innovation (QAAFI), The University of Queensland, 306 Carmody Road, Brisbane, Queensland 4072, Australia; Queensland Alliance for Agriculture and Food Innovation (QAAFI), The University of Queensland, 306 Carmody Road, Brisbane, Queensland 4072, Australia; Queensland Alliance for Agriculture and Food Innovation (QAAFI), The University of Queensland, Hermitage Research Facility, 604 Yangan Road, Warwick, Queensland 4370, Australia

**Keywords:** Auxin efflux carriers, drought adaptation, PIN-formed, plant architecture, sorghum, stay-green

## Abstract

Plant architecture modification (e.g. short-stature crops) is one of the key outcomes of modern crop breeding for high-yielding crop varieties. In cereals, delayed senescence, or stay-green, is an important trait that enables post-anthesis drought stress adaptation. Stay-green crops can prolong photosynthetic capacity during grain-filling period under post-anthesis drought stress, which is essential to ensure grain yield is not impacted under drought stress conditions. Although various stay-green quantitative trait loci have been identified in cereals, the underlying molecular mechanisms regulating stay-green remain elusive. Recent advances in various gene-editing technologies have provided avenues to fast-track crop improvement, such as the breeding of climate-resilient crops in the face of climate change. We present in this viewpoint the focus on using sorghum as the model cereal crop, to study PIN-FORMED (*PIN*) auxin efflux carriers as means to modulate plant architecture, and the potential to employ it as an adaptive strategy to address the environmental challenges posed by climate uncertainties.

## Teaser

Stay-green is a valuable trait in crop breeding that enables plants to remain photosynthetically active longer and delay senescence during grain filling under post-anthesis drought stress. In sorghum, the expression of stay-green is a consequence of regulation of the water supply and demand framework through modulation of plant canopy and root architecture. Several studies have identified members of the PIN-FORMED (PIN) family of auxin efflux carriers as potential candidate genes that can modulate plant architecture to express stay-green sorghum. Using sorghum as the model species, further research on PIN auxin efflux carriers as a means to modulate plant architecture holds the potential to employ it as an adaptive strategy for crop breeding to address the environmental challenges pose by climate uncertainties.

## Introduction

Climate change directly affects food systems, reducing food and water security, while slowing the growth of agricultural productivity for the past 50 years globally (IPCC 2022). The scale of potential negative impacts of climate change on crop production worldwide has been highlighted in several recent analyses. Although there are some positive impacts of climate change such as CO_2_ fertilization (Long *et al.* 2006), the negative impacts dominate (Lobell and Field 2007; Lobell and Gourdji 2012; Guan *et al.* 2017; Zhao *et al.* 2017). Ortiz-Bobea *et al.* (2021) indicated that anthropogenic climate change has reduced global agricultural total factor productivity by about 21 % since 1961, a slowdown that is equivalent to losing the last 7 years of productivity growth. There is high agreement that global crop production is becoming more vulnerable to ongoing climate change since the pre-industrial era (Ortiz-Bobea *et al.* 2021; IPCC 2022).

The Green Revolution has greatly increased the world’s food supplies by introducing various high-yielding crops, especially wheat and rice, while simultaneously increasing cropping intensity, and improving cropping and irrigation systems (Evenson and Gollin 2003; Pingali 2012; Brainerd and Menon 2014; Somvanshi *et al.* 2020). These high-yielding crops share a common plant architecture trait: reduced plant height. The reduced plant height is attributed to the genes involved in the gibberellic acid (GA) signalling pathway, in which the varieties carrying the GA-insensitive alleles do not increase in plant height with increased growth, resulting in increased partitioning of resources to the grain (Peng *et al.* 1999; Sasaki *et al.* 2002; Hedden 2003; Thomas 2017). However, plant architecture modification via the perturbations to GA signalling also presents adverse effects on developmental processes such as early seed vigour and coleoptile growth (Botwright *et al.* 2001; Rebetzke *et al.* 2001), which are crucial traits for cultivating crops in water-limited environments. Therefore, the adoption of these varieties is more suitable for favourable environments in which inputs such as fertilizer, pesticides and adequate irrigation are required. Adopting these high-yielding varieties at the expense of the environment is not a sustainable option for the future of crop production. Furthermore, the negative effects of climate change would create more climate uncertainties in many agriculture lands globally. More sustainable approaches would be required to safeguard the future of crop production and food security.

Crop production in water-limited environments is dependent on the capacity of the crop to favourably balance water supply and demand. Understanding the interactions among genetics (G), management (M) and environment (E) to optimize G × E × M strategies for production in water-limited environments is critical. One of the most significant challenges facing crop improvement programmes globally is the capacity to adequately match crop production with demand, thereby ensuring food security in the face of climate change. To achieve this end, genetic resources must be better explored (or alternatively created by technologies such as gene editing) to reveal molecular mechanisms that increase developmental plasticity in traits driving water supply and demand, thereby enabling sessile plants to adapt more readily to a hotter and drier world. Other important factors such as the environment and crop management practices also contribute significantly to crop production. Therefore, climate prediction studies on the effect of climate change on various cropping systems and the employment of optimal crop management practices are important for the risk management of crop production (Challinor *et al.* 2009; Lehmann *et al.* 2013; Zhao *et al.* 2017; Hasegawa *et al.* 2022).

This nexus of climate change, decreased agricultural productivity, food insecurity and water scarcity led to the inevitable conclusion that climate-smart crops and cropping systems, as well as their supply chains, need to be more resilient in the face of these challenges. In this viewpoint article, we emphasize the use of sorghum (*Sorghum bicolor*) as the model cereal crop, together with the available genetic resources on drought adaptation and the stay-green trait, to a small extent realistically, addresses one component of this overall challenge. We describe the connections between plant architectural traits, water uptake, the stay-green trait and the underlying identified gene candidates in sorghum, while at the molecular level, we highlight the extent of the PIN-FORMED (*PIN*) gene family members that can modulate processes in plants that affect components of both water supply (e.g. root architecture) and demand (e.g. canopy development), as potential targets for improving crop yield and resilience to environmental unpredictability. The critical point here is that adaptation mechanisms operating at the molecular level must scale up to the crop level, resulting in enhanced biomass and grain yield in the field under drought conditions.

## Plant Architectural Traits Influencing Water Uptake and Transport Through Plants

Water uptake in plants is affected by the soil–plant–atmosphere continuum, which is controlled by the shoots and roots (Blum 2011). The shoot and root systems are closely linked as each contributes to their overall functionality, maintenance and survival. Products of photosynthesis are needed by the roots for their growth and maintenance, while water and nutrient uptake from roots are required in various biological processes taking place in parts of the plant above ground (Topp and Benfey 2012). Plant hormone signalling in response to environmental stimuli also takes place between the shoot and root systems (van Norman *et al.* 2004; Umehara *et al.* 2008).

Various plant morphological features have been identified to assist in balancing plant growth and survival under conditions when stresses occur. Morphological features, such as leaf wax cuticles, smaller leaf area, reduced canopy size and tillering, have been shown to assist in drought stress adaptation (Ristic and Jenks 2002; Gómez-Del-Campo *et al.* 2003; Borrell *et al.* 2014*a*). Root architecture, such as root length density, root surface area and root–soil contact, also play an important role in the water extraction efficacy (Kramer and Boyer 1995; Schwinning *et al.* 2005; Saidi *et al.* 2010; White and Kirkegaard 2010).

In addition to the structure and size of plant organs that could contribute to drought adaptation, the functionality of the canopy and root also play crucial roles in determining hydraulic conductance throughout the plant, how roots contribute to water extraction and the timing of water uptake (Tardieu 2005; Vadez *et al.* 2013; Vadez 2014; Tardieu *et al.* 2018). Canopy traits involved in the control of water movement through the plant include leaf venation (Nardini *et al.* 2003; Scoffoni *et al.* 2011; Sack and Scoffoni 2013), mesophyll anatomy (Brodribb *et al.* 2010; Buckley *et al.* 2015; John *et al.* 2017) and stomatal dynamics (Farquhar *et al.* 2002; Buckley 2005), while root traits include root permeability and root–mycorrhizal symbiotic relationship (McCully 1999; Finlay 2008; Bárzana *et al.* 2012). It is important to note that many traits are often interrelated through deterministic relations (Vadez *et al.* 2011*a*, *b*). For example, in the case of stay-green trait, which is associated with delayed senescence of the plant, it can share quantitative trait loci (QTLs) associated with plant growth, transpiration, drought adaptation and allometric relations (Vadez *et al.* 2011*b*). It is important to note that the discovery of molecular mechanisms that control a specific aspect of development (e.g. cell expansion, flowering time, etc.) only offers a simplistic view of how that development is regulated, and not the bigger picture that involves other genetic components from other genetic pathways, including the environment factors.

## The Stay-Green Trait and its Underlying Complex Pathway Interactions

Stay-green can be broadly defined as a heritable trait in which the plants display prolonged greenness and delayed senescence in the vegetative tissues (i.e. leaves and stem) relative to plants that do not have this trait (Thomas and Ougham 2014). Stay-green can be categorized into functional and cosmetic types. Cosmetic stay-green generally refers to features caused by defects in the chloroplast catabolism pathway, which can be beneficial to certain specialty crops such as forage or turf grass species (Myers *et al.* 2018), but is not associated with maintenance of photosynthesis. In contrast, functional stay-green refers to the retention of photosynthetic capacity for longer under post-anthesis drought stress, resulting in sustained biomass production and grain yield (Jordan *et al.* 2012; Borrell *et al.* 2014*b*; Christopher *et al.* 2016). Plants with functional stay-green exhibit traits that (i) reduce canopy size (e.g. reduced tillering, smaller upper leaves), thereby shifting water use from pre- to post-anthesis (Borrell *et al.* 2014*a*, *b*; George-Jaeggli *et al.* 2017), and (ii) increase water uptake throughout crop growth (e.g. narrow root angle) (Borrell *et al.* 2014*a*, *b*). These functional adaptations would be modulated by gene families that regulate branching and organ size in plants (Borrell *et al.* 2022).

Kassahun *et al.* (2010) demonstrated that partial introgression of stay-green QTLs from the stay-green donor parent B35 into the senescent background R16 produced higher leaf chlorophyll content both before and during leaf senescence, reduced leaf senescence and higher relative grain yield in two of the three post-anthesis drought environments tested. In field studies using near-isogenic lines (NILs) consisting of individual stay-green (*Stg*) QTLs of *Stg1*, *Stg2*, *Stg3* and *Stg4* from the stay-green line of Tx642 (also known as B35) have also shown that *Stg* QTLs affect crop water uptake patterns, shifting crop water use from the pre- to post-anthesis period, at high and low planting densities (Borrell *et al.* 2014*b*). Shifting crop water use to the grain-filling period is a consequence of the smaller canopy size in the *Stg* NILs before anthesis (Borrell *et al.* 2014*a*, *b*; George-Jaeggli *et al.* 2017). This shift in crop water use from pre- to post-anthesis period is important to ensure water availability during grain filling, which can greatly increase grain yield (Hammer 2006; Manschadi *et al.* 2006; Kirkegaard *et al.* 2007). This suggests that the expression of stay-green is likely the consequence of regulated plant organ development controlled by the introgressed genes within these stay-green QTL regions. Characterizing and studying the functions of these genes within the stay-green QTL will be essential to decipher the molecular mechanisms that confer stay-green.

Water and nitrogen dynamics are important components of the stay-green trait in sorghum. However, increased water uptake during the grain-filling period in stay-green genotypes is likely the key driver of enhanced post-anthesis nitrogen uptake. Hence, the regulation of stay-green is associated with nitrogen dynamics that revolve around the nitrogen supply and demand framework (Borrell and Hammer 2000; Borrell *et al.* 2001; van Oosterom *et al.* 2010*a*, *b*), although these dynamics are largely regulated by water availability in water-limited environments. There is also some evidence that stay-green enhances water use efficiency (Vadez *et al.* 2011*a*, *b*, 2014; Kholová *et al.* 2014). Therefore, the physiological basis of stay-green in sorghum is relatively well understood. However, the underlying molecular mechanisms that regulate stay-green remain poorly understood. The stay-green phenotype is an emergent consequence of processes that regulate water supply (e.g. root architecture) and demand (e.g. canopy development), and is, therefore, a complex trait. Hence, the functionality of stay-green is the product of interactions and crosstalk between proteins of various biological and developmental processes. The expression of stay-green is also controlled by physiological changes that are regulated by developmental processes involving phytohormones and other environmental signalling proteins.

## Auxin, Plant Development and PIN Auxin Efflux Carriers

**Figure 1.**
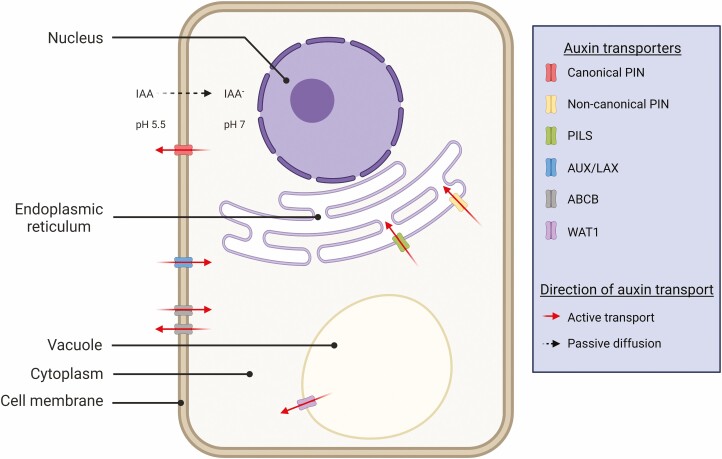
The localization of various auxin transporters: canonical and non-canonical PINs, PILS, AUXIN1/LIKE-AUX1 (AUX/LAX), ATP-BINDING CASSETTE SUBFAMILY B (ABCB) and WALLS ARE THIN1 (WAT1) and the direction of auxin flow in a plant cell. The cell wall, apoplast and other cell components where auxin transporters have not been reported to be localized at are excluded from the figure. Figure template was adapted from ‘Structural Overview of a Plant Cell’, by BioRender.com (2022). Retrieved from https://app.biorender.com/biorender-templates, with modifications.

The phytohormone, auxin, drives many aspects of plant development, and many of these processes are dependent on the perception, distribution and biosynthesis of auxin in the cells and tissues. Auxin transport naturally dictates the flow and distribution of auxin, whereby auxin is perceived as signals in the cell, binding to a range of receptor proteins and triggering a cascade of processes involving the synthesis of various auxin-responsive proteins (Ulmasov *et al.* 1999; Gray *et al.* 2001; Tan *et al.* 2007; Chapman and Estelle 2009; Boer *et al.* 2014). In the auxin transport pathways, the PIN auxin efflux carriers, AUXIN1/LIKE-AUX1 (AUX/LAX) auxin influx carriers and other auxin-related transporters such as ATP-BINDING CASSETTE SUBFAMILY B (ABCB) auxin transport proteins, PIN-LIKES (PILS) and WALLS ARE THIN1 (WAT1) are essential in regulating the cell-to-cell transport of auxin, maintaining auxin gradients and intracellular auxin homeostasis (Noh *et al.* 2001; Barbez *et al.* 2012; Péret *et al.* 2012; Ranocha *et al.* 2013; Balzan *et al.* 2014) (Fig. 1).

**Figure 2.**
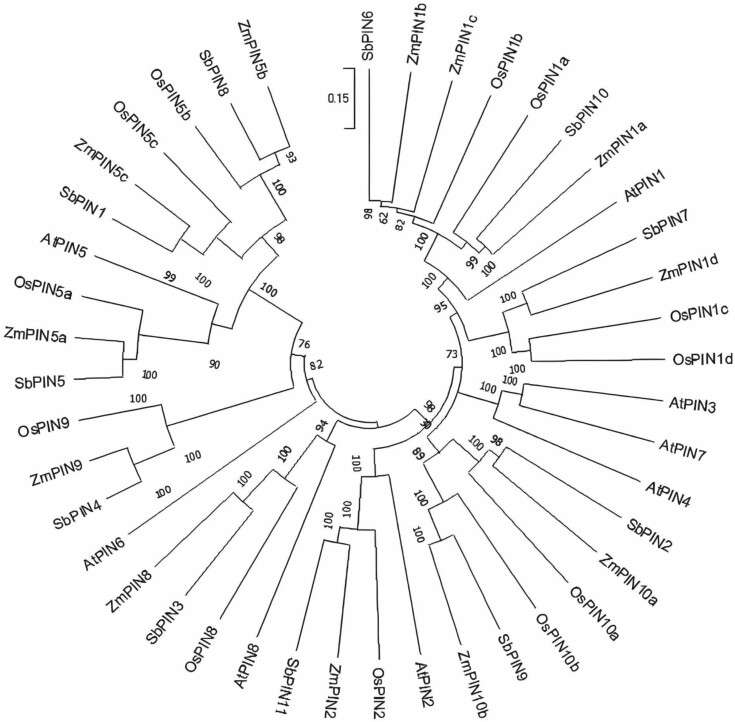
The phylogeny tree showing PIN members from Arabidopsis (At), maize (Zm), rice (Os) and sorghum (Sb). The evolutionary history was inferred using the neighbour-joining method (Saitou and Nei 1987). The optimal tree is shown. The percentage of replicate trees in which the associated taxa clustered together in the bootstrap test (1000 replicates) are shown next to the branches (Felsenstein 1985). The tree is drawn to scale, with branch lengths in the same units as those of the evolutionary distances used to infer the phylogenetic tree. The evolutionary distances were computed using the Poisson correction method (Zuckerkandl and Pauling 1965), and are in the units of the number of amino acid substitutions per site. This analysis involved 43 amino acid sequences. All ambiguous positions were removed for each sequence pair (pairwise deletion option). There are a total of 919 positions in the final dataset. Evolutionary analyses were conducted in MEGA11 (Tamura *et al*. 2021).

PIN proteins are categorized as canonical and non-canonical based on the presence and absence of four highly conserved motifs in the central hydrophilic loop (Bennett *et al.* 2014). Most canonical PINs across various plant species have long central hydrophilic loops, and the non-canonical PINs have shorter central hydrophilic loops (Křeček *et al.* 2009; Viaene *et al.* 2013; Bennett *et al.* 2014). Canonical and non-canonical PINs are generally located at the plasma membrane and endoplasmic reticulum, respectively (Balzan *et al.* 2014) (Fig. 1). Canonical PINs are involved in the efflux transport of auxin from the cytoplasm to the extracellular environment, whereas non-canonical PINs are thought to contribute to intracellular auxin distribution and homeostasis (Zažímalová *et al.* 2010) (Fig. 1). The number of PIN family members differs among different plant species. In Arabidopsis (*Arabidopsis thaliana*), there are 8 different PINs (AtPIN1–AtPIN8), whereas there are 11 different PINs (SbPIN1–SbPIN11) in sorghum (Clouse and Carraro 2014). Within monocots, rice and maize also contain PIN family members that are monocot-specific (Wang *et al.* 2009; Forestan *et al.* 2012; Balzan *et al.* 2014). Phylogenetic tree analysis using PINs from Arabidopsis, rice, maize and sorghum shows that SbPIN2, SbPIN4 and SbPIN9 fall in the group of monocot-specific PINs (Fig. 2).

PINs are mainly polar localized in the cell, enabling polar transportation of auxin and the formation of auxin gradients in plants. PINs regulate auxin distribution in plants, which in turn regulate various developmental processes, such as branching in a wide range of crop species (Beveridge 2000; Ishikawa *et al.* 2005; Simons *et al.* 2006; Gallavotti *et al.* 2008). Functional analysis studies in Arabidopsis have shown that disruption in the expressions of *PIN*s can cause abnormality in developmental processes such as floral formation, patterning of the root and root gravitropism (Okada *et al.* 1991; Müller *et al.* 1998; Friml *et al.* 2002*a*, *b*). In rice, perturbations to the expressions of *OsPIN3t*, also referred to as *OsPIN10a* (Wang *et al.* 2009), can affect adventitious root emergence and development (Zhang *et al.* 2012). Plant architectural traits such as tiller number and shoot height can also be affected by altering the expressions of *OsPIN1b* and *OsPIN2* (Xu *et al.* 2005; Chen *et al.* 2012). Since PINs are integral in establishing and maintaining the auxin gradients in cells and tissues, thus determining the development of the plant, it is logical to suggest that differences in the expression of various PINs may result in various plant phenotypes and architectures.

In monocots such as rice and maize, *PIN* genes have been shown to be expressed in specific tissues at various developmental stages (Miyashita *et al.* 2010; Forestan *et al.* 2012; Li *et al.* 2019), demonstrating the relationship between *PIN* gene expression in plant organs and architecture development (both above- and below-ground). In sorghum, the expression of *SbPIN* genes has been reported in several expression studies (Shen *et al.* 2010; McCormick *et al.* 2018), and changes in plant architecture such as tillering, leaf size and root traits associated with increased *SbPIN* gene expression are first demonstrated in Borrell *et al.* (2022). While the current literature provides examples of how the *PIN* gene family modulates branching and floral architecture in Arabidopsis, as well as some functional studies on model crops such as rice, there is a paucity of literature on how these genes might impact drought adaptation at the molecular level, and even less about how they might contribute to climate-resilience at the crop level.

## Connecting Plant Architecture, Stay-Green and *PIN* Genes in Sorghum

A large number of QTLs contributing to the stay-green phenotype in sorghum have been identified (Crasta *et al.* 1999; Subudhi *et al.* 2000; Tao *et al.* 2000; Xu *et al.* 2000; Kebede *et al.* 2001; Haussmann *et al.* 2002; Srinivas *et al.* 2009; Sabadin *et al.* 2012; Rama Reddy *et al.* 2014; Wang *et al.* 2014; Sukumaran *et al.* 2016). Both characterized and uncharacterized genes in these QTL regions have the potential to alter plant architecture via various pathways and mechanisms that confer stay-green. However, this would require detailed gene functional studies of these lines. Plant architecture modifications via the introgression of the stay-green QTLs of *Stg1*, *Stg2*, *Stg3* and *Stg4* from the stay-green line of Tx642 into the senescent line of Tx7000 suggested potential candidate genes within these regions as key regulators of plant organ development (Borrell *et al.* 2014*a*, *b*; George-Jaeggli *et al.* 2017). Genome analysis of these QTLs has identified 9 out of 11 members of the PIN auxin efflux carriers as potential candidates that can modulate plant architecture to express the stay-green phenotype (Borrell *et al.* 2009, 2015, 2018; Mace *et al.* 2019). This has resulted in the identification of *SbPIN1*, *SbPIN2* and *SbPIN4* as key candidate genes within the stay-green QTLs of *Stg3b*, *Stg2* and *Stg1*, respectively.

The fact that *PIN* genes have been shown to be involved in plant development and physiological processes, such as branching, leaf vein patterning, inflorescence development, root development and gravitropism in various plants (Okada *et al.* 1991; Müller *et al.* 1998; Blilou *et al.* 2005; Xu *et al.* 2005; Ding *et al.* 2012; Cazzonelli *et al.* 2013; Sawchuk *et al.* 2013; Lu *et al.* 2015), further suggests the importance of understanding how *SbPIN1*, *SbPIN2* and *SbPIN4* underpin the significant stay-green QTLs of *Stg3b*, *Stg2* and *Stg1*, respectively. Furthermore, the use of single *Stg* QTL NILs of *Stg1* (containing *SbPIN4*), *Stg2* (containing *SbPIN2*) and *Stg3b* (containing *SbPIN1*) has demonstrated the contribution of each QTL in conferring the stay-green phenotypes (Borrell *et al.* 2014*a*, *b*). However, it is also possible that the emergent phenotypes of these *Stg* NILs could be attributed to effects from the transcription of other genes located within the QTLs, together with any regulatory cues that could be different in the stay-green line Tx642 relative to the introgressed background lines.

## Future Directions in Plant Architecture Designs for Crop Adaptation to Climate Change

**Figure 3.**
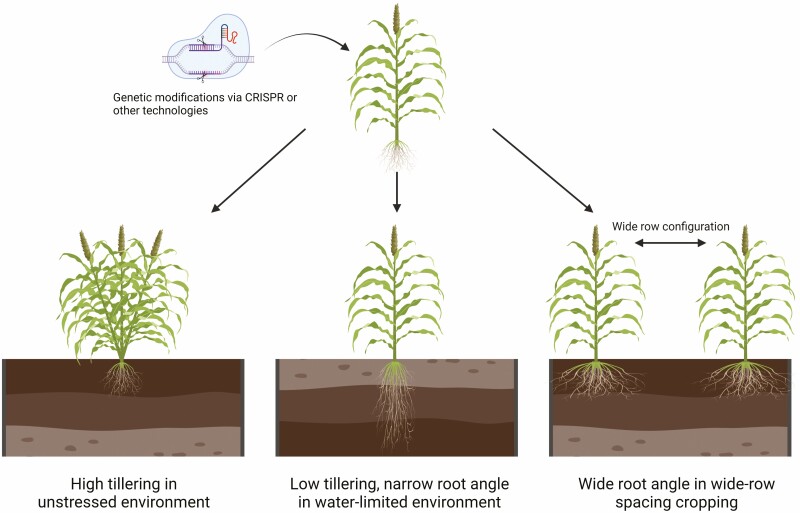
The potential cereal ideotypes for different cultivation scenarios can be created from the understanding and alteration of PINs in sorghum. Created with BioRender.com.

Developmental plasticity can significantly improve yield stability in agriculture. For example, Borrell *et al.* (2022) demonstrated that differences in the expression of *SbPIN2* can significantly increase or decrease canopy size via differences in tillering and/or individual leaf size. Hence, modulating the expression of *SbPIN2* can produce a wide range of tillering phenotypes. It is hypothesized that the alteration of *SbPIN2* expression may also induce other physiological changes in the panicle architecture, leaf parameters such as leaf vein density and photosynthesis, and root architecture parameters such as nodal root angle and root biomass distribution per soil depth. If this is the case, then the expression of *SbPIN* genes could be modulated to affect the above- and below-ground architecture of sorghum and, ultimately, enable the development of climate-resilient crops via the modification of traits that determine water supply (e.g. root architecture) and demand (e.g. canopy development) to suit various cultivation scenarios (Fig. 3).

Plants are remarkably plastic, such that a single genotype can exhibit multiple phenotypes, depending on the environment and management scenarios in which they are grown. Therefore, a low-tillering genotype could be sown at a range of densities to create multiple G × M scenarios for grain-growers. Low-tillering genotypes could be grown under low plant densities in more stressed environments or grown under medium plant densities in moderately stressed environments. Alternatively, high-tillering genotypes could be grown in regions where water is not a major limitation. In these environments, larger canopies can intercept more radiation, producing higher biomass and grain yield. Canopy modification could also be combined with manipulation of root architecture. Genotypes exhibiting narrower or wider root angle can be adopted for different water extraction scenarios, in which narrower root angle genotypes allow more water extraction at depth during end-of-season drought, while wider root angle genotypes could be beneficial for extracting water from between rows in wide-row spacing configurations (Fig. 3). Such a strategy would enable grain-growers to create specific canopy size and root architecture combinations via G × M manipulation to match the water status of their farm (e.g. the amount of stored soil moisture in the field) and the predicted rainfall (e.g. medium-term weather forecast).

The co-location of *SbPIN* genes with major stay-green QTLs in sorghum, coupled with the roles of PINs in plant development and physiological processes reported in various plants, suggest that differential expression and post-translational modifications to *SbPIN* genes could be the contributing factor to stay-green. However, it is also important to note that other genes located within the *Stg* QTL regions studied in sorghum, both characterized and uncharacterized, have the potential to alter plant architecture via various pathways and mechanisms that confer stay-green. Despite the potential regulatory challenges in gene-editing and difficulties working with recalcitrant crops such as sorghum (Massel *et al.* 2021; Wong *et al.* 2022), the combination of the two approaches of (i) expressing *SbPIN*s attached to plant tissue-specific promoters and (ii) editing regulatory regions or the coding sequences of *SbPIN*s through CRISPR/Cas9 technology should be a good strategy for designing future crops with desirable plant architectures to overcome climate change. Experiments could also include commercial lines with various tillering genotypes to act as controls and checks. Simulated models such as the Agricultural Production Systems sIMulator could also be tested, however, fundamental physiological studies on traits via the manipulation of these *SbPIN*s would need to be documented first so that it could be used as inputs for simulation models. Successful integration of these approaches into crop production systems will also require field-scale physiological studies and the validation of G × M × E interactions.

## Conclusion

The connection between stay-green and the regulation of plant growth and architectural development through possible interactions with *SbPIN* genes is a very intriguing concept. The connection has arisen from the discovery that 9 of 11 *PIN* genes in sorghum are co-located with stay-green QTL, and that manipulation of *PIN* genes can cause a stay-green phenotype via modification of sub-traits that impact components of water supply (e.g. root architecture) and water demand (e.g. canopy development). Evidence from phenotyping studies on *SbPIN* overexpression lines in sorghum show how plant architecture from the organ scale to the whole-plant scale can be modified via alterations of *PIN* genes (Borrell *et al.* 2022), providing additional impetus to hypothesize that differential expression and post-translational modifications to *SbPIN* genes could be the contributing factor to stay-green.

Despite the plethora of studies on the regulation and function of *PIN* genes in model dicots and monocots, understanding the regulation and function of *SbPIN* genes in sorghum is relatively lacking. Hence, in-depth studies of the regulation and function of *SbPIN* genes in modulating plant architecture in sorghum are warranted. Functional analysis of *SbPIN* genes using molecular techniques and technologies such as CRISPR/Cas9 should complement the abundance of established stay-green physiological, QTL association and modelling studies in sorghum, providing a more complete view on the effects of *SbPIN* gene expression on the modulation of plant architecture, and the interactions of G × E × M. We hypothesize that the mechanisms involving *PIN* genes regulating canopy development, and possibly root architecture and grain yield, also function similarly in other major cereal crops to enhance productivity under drought conditions. Evaluating the impacts of *PIN* genes on canopy, root and panicle development in major cereal crops would shed light on the practicality of targeting these genes for designing climate-resilient cereal ideotypes.

## Data Availability

No new data were generated or analysed in support of this manuscript.

## Notes

**Phenome, Genome & Environment. Chief Editor:** Colleen Doherty
